# In Response

**DOI:** 10.4269/ajtmh.2011.10-0584b

**Published:** 2011-02-04

**Authors:** Sharon Peacock, Paul Newton

**Affiliations:** University of Cambridge,; Cambridge, United Kingdom; E-mail: sp10@sanger.ac.uk; Wellcome Trust-Mahosot Hospital-Oxford; Tropical Medicine Research Collaboration; Vientiane, Laos

Dear Sir:

We thank Cohen and others^1^ for their commendation of the article by Moore and others^2^ on pneumococcal serotype distribution in Laos. We concur with their comment that there is a paucity of data on pneumococcal serotypes causing invasive disease in this region of the world. Real-time polymerase chain reaction assays to enhance the serotype determination of pneumococci in cerebrospinal fluid (CSF), such as the one we have developed, are potentially important new tools with particular use where frequent over the counter antibiotic use before hospital presentation reduces the yield of culture. The study by Moore and others^2^ reported the pneumococcal serotype associated with invasive pneumococcal disease (IPD) in 33 patients, but the effort required to obtain this information was large and included blood culture from 10,799 unselected febrile patients and 353 CSF cultures. We agree with Cohen and others^1^ when they say that what is missing in much of the developed world right now is high quality surveillance rather than high-quality vaccines. An important lesson to be reiterated by our study, however, is that defining the impact of the introduction of pneumococcal vaccines in Laos must rely in large part on clinical measurement of the overall reduction in rates of IPD.
